# Bilateral renal metastasis following radical prostatectomy for prostate cancer: a case report

**DOI:** 10.3389/fmed.2026.1718567

**Published:** 2026-05-29

**Authors:** Qiu Ming He, Jun Jie Tang, Chao Hao, Yi Jiang, Ji Huang, Jian Biao Huang, Xin Hua Tu

**Affiliations:** Jiangxi Clinical Research Center for Cancer, Jiangxi Cancer Hospital and Institute, The Second Affiliated Hospital of Nanchang Medical College, Nanchang, China

**Keywords:** endocrine therapy, hepatic metastasis, monitoring, prostate cancer, renal metastasis

## Abstract

This article reports the diagnosis and treatment of an 81-year-old man with advanced prostate cancer presenting with multiple organ metastases. The patient had undergone laparoscopic radical prostatectomy 5 years prior and received intermittent postoperative endocrine therapy with leuprorelin acetate and bicalutamide. He presented with hematuria, and laboratory evaluation revealed a markedly elevated prostate-specific antigen level (total PSA 274.71 ng/mL, free PSA 63.50 ng/mL). Contrast-enhanced CT demonstrated bilateral renal masses, retroperitoneal lymphadenopathy, and liver metastases. A biopsy of the right renal lesion confirmed metastatic prostate acinar adenocarcinoma, with immunohistochemistry showing PSA (+), Ki67 (40%, indicating high proliferative activity), and succinate dehydrogenase subunit B (SDHB) (+). Following a change in the endocrine therapy regimen to rezvilutamide combined with degarelix, the patient’s hematuria significantly improved. This case highlights the critical importance of rigorous postoperative surveillance and timely, standardized endocrine therapy in prostate cancer management. It also contributes to the limited literature on visceral metastases in castration-sensitive prostate cancer (HSPC) and underscores the need for further exploration of optimal treatment strategies for advanced metastatic disease.

## Introduction

Prostate cancer remains one of the most common malignancies affecting men globally. While bone metastases represent the predominant form of disease dissemination, visceral metastases occur relatively infrequently, documented in approximately 10–15% of patients with advanced disease (1, 2). The introduction of novel endocrine therapies, including next-generation androgen receptor pathway inhibitors such as abiraterone and enzalutamide, has substantially improved survival outcomes for patients with metastatic prostate cancer (3). However, these treatment advances have simultaneously altered disease progression patterns, leading to the emergence of heterogeneous responses where some lesions regress while others progress, and unusual metastatic sites may develop (4). This evolving clinical phenomenon, often described as “treatment-driven evolution,” highlights the necessity to reconsider traditional progression paradigms and adapt management strategies accordingly (5).

We present an instructive case of multi-organ visceral metastasis involving both kidneys and the liver, an exceptionally rare presentation in the contemporary era of modern hormonal therapies. Through this case illustration, we examine the clinical characteristics, radiographic features, and therapeutic decision-making processes associated with heterogeneous disease progression. Furthermore, we review existing evidence regarding optimal sequencing of systemic therapies and explore the potential role of local interventions in managing oligometastatic visceral disease (6).

By synthesizing clinical imaging findings, histopathological assessment, and molecular profiling data were available, this analysis aims to deepen the understanding of the evolving landscape of metastatic prostate cancer. Additionally, it emphasizes the importance of personalized treatment strategies when confronting atypical metastatic patterns, particularly in the context of a dissociated response to modern therapies. The insights derived from this case contribute to the growing body of literature on treatment-emergent metastatic patterns and their clinical management in the era of precision oncology.

## Case presentation

An 81-year-old man with a history of prostate cancer diagnosed 5 years prior was admitted due to persistent hematuria. The patient underwent laparoscopic radical prostatectomy on 11 September 2018, following 3 months of neoadjuvant endocrine therapy. Histopathological examination of the surgical specimen revealed prostatic acinar adenocarcinoma with perineural invasion and a Gleason score of 4 + 4 = 8. Postoperatively, adjuvant androgen deprivation therapy (ADT) was initiated in October 2018, consisting of leuprorelin acetate and bicalutamide. After 1 year of treatment, ADT was discontinued in October 2019. Subsequently, the patient was lost to regular follow-up, with no consistent monitoring of PSA levels. At presentation in March 2025, the patient’s medical history included hypertension controlled with amlodipine and no diabetes. Family history was negative for prostate, breast, or colorectal cancer in first-degree relatives. Physical examination revealed blood pressure 138/84 mmHg, heart rate 82 bpm, ECOG performance status 1, no palpable abdominal masses, no costovertebral angle tenderness, and the digital rectal examination was not repeated due to prior prostatectomy. On 26 March 2025, the patient presented with persistent hematuria. Laboratory investigations revealed a markedly elevated total PSA level of 274.71 ng/mL. Whole-body bone scintigraphy showed no evidence of skeletal metastases. However, contrast-enhanced abdominal CT demonstrated bilateral renal masses, retroperitoneal lymphadenopathy, and multiple liver metastases (Figure 1). An ultrasound-guided biopsy of the right renal mass was performed, and histopathological examination confirmed metastatic prostatic acinar adenocarcinoma. Immunohistochemical staining was positive for PSA, showed a Ki-67 proliferation index of 40%, and was positive for SDHB (Figure 2). In April 2025, following confirmation of disease progression, the treatment regimen was adjusted to rezvilutamide (240 mg orally once daily) in combination with degarelix (initial dose 240 mg subcutaneously, followed by maintenance doses of 80 mg every 4 weeks). The patient’s hematuria resolved significantly following the initiation of the new regimen, and he remains under active surveillance.

**Figure 1:**
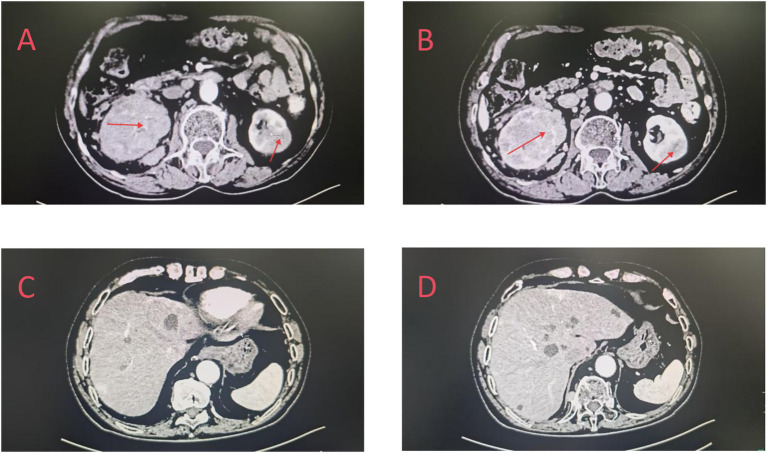
Abdominal CT reveals metastatic lesions in both kidneys **(A,B)** and the liver **(C,D)**. Two kidneys, multiple occupying lesions in the right renal area, retroperitoneum, the indicated diaphragmatic foot area, multiple enlarged lymph nodes on the right side of the posterior mediastinum and the spine, considered as possible metastasis: Some nodules in the liver show mild enhancement, considered as metastasis.

**Figure 2:**
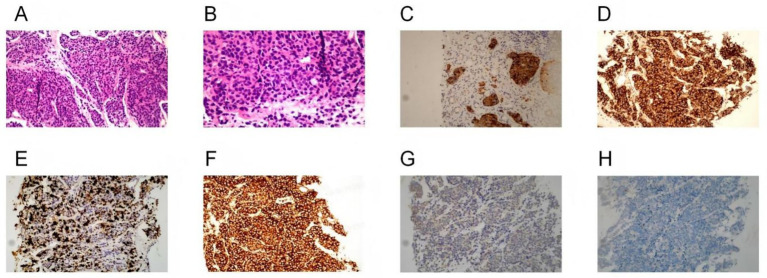
Hematoxylin and eosin–stained pathological images of a renal metastatic tumor biopsy **(A,B)**. Immunohistochemical staining with PSA antibody **(C,D)** demonstrates positive PSA expression. Immunohistochemical staining with Ki-67 antibody **(E)** shows positive Ki-67 expression. Immunohistochemical staining with SDHB antibody **(F)** reveals positive SDHB expression. Synaptophysin (Syn) immunohistochemical staining **(G)** shows negative Syn expression. Chromogranin A (CGA) immunohistochemical staining **(H)** indicates negative CGA expression.

## Discussion

Renal metastasis is a rare occurrence in prostate cancer, with previous literature reporting an incidence of less than 3% (5). Unlike the more common bone and lymph node metastases, parenchymal renal metastasis often signifies that the disease has entered an advanced stage (6). In this case, the patient’s presentation of bilateral renal metastasis accompanied by liver metastasis is particularly unusual. Its mechanism may be related to tumor clonal evolution driven by irregular treatment exposure (7). Notably, the absence of detectable bone metastases despite extensive visceral involvement represents an atypical metastatic pattern (8).

The immunohistochemical profile in our case provides crucial insights into the tumor biology. In response to the potential concern of treatment-emergent neuroendocrine differentiation, we performed additional staining, which was negative for synaptophysin and chromogranin A. However, the tumor cells showed preserved SDHB expression, arguing against high-grade neuroendocrine carcinoma with SDHx deficiency (9). This profile (PSA-positive, neuroendocrine marker-negative, SDHB-positive) supports an aggressive adenocarcinoma phenotype rather than canonical treatment-emergent neuroendocrine prostate cancer. The preserved SDHB expression suggests alternative resistance mechanisms through metabolic adaptations, potentially involving enhanced aerobic glycolysis and mitochondrial oxidative phosphorylation (10). The mechanisms underlying such extensive visceral metastasis likely involve complex interactions between cancer stem cell alterations, epithelial-mesenchymal transition, and microenvironment remodeling (11). The high Ki67 proliferation index of 40% indicates highly aggressive tumor behavior, possibly reflecting the emergence of castration-resistant clones under the selective pressure of irregular treatment (12).

Regarding therapeutic strategy, the selection of rezvilutamide over conventional agents was based on its novel structure and potentially broader efficacy against resistant disease (13). The combination with degarelix was strategic, as this GnRH antagonist provides immediate testosterone suppression without the initial surge associated with agonists (14). This rapid-onset combination aims to achieve swift disease control in high-burden scenarios (15). The subsequent resolution of hematuria supports the regimen’s palliative benefit, though long-term outcomes in such advanced disease remain concerning.

In conclusion, this case highlights several clinically noteworthy aspects of disease progression in hormone-sensitive prostate cancer (HSPC). First, it presents an unusual metastatic pattern characterized by predominant visceral involvement, including the rare occurrence of renal metastases, in the complete absence of osseous lesions. This distribution diverges from the typical osteotropic nature of prostate cancer and suggests that atypical presentations should be considered during biochemical recurrence. Second, the immunohistochemical profile observed in this case (PSA+, Ki67 40%, SDHB+) is reported, although the absence of additional renal-exclusion markers (e.g., PAX8) and molecular profiling data limits the ability to draw firm conclusions regarding metabolic adaptation versus neuroendocrine differentiation. Third, we provide longitudinal PSA data (baseline 274.71 ng/mL, 142.3 ng/mL at 4 weeks, and 59.8 ng/mL at 12 weeks) and corresponding imaging follow-up, which demonstrate a discordant but favorable response to rezvilutamide plus degarelix. Finally, given the limitations of a single case report, the findings should be interpreted as hypothesis-generating rather than definitive. Further studies with larger patient populations and comprehensive molecular characterization are needed to elucidate the biological mechanisms underlying visceral metastases in HSPC and to optimize treatment strategies for patients with non-canonical patterns of progression.

## Conclusion

Metastases to bilateral kidneys and liver post-prostatectomy signify highly aggressive disease, potentially from treatment-irregularity-induced clonal evolution. These cases carry poor prognoses and require aggressive multimodal therapy. Our findings demonstrate that novel endocrine therapies retain symptom control value for visceral metastases. Given their rarity, more clinical evidence and molecular investigations are crucial to optimize management strategies.

## Data Availability

The original contributions presented in the study are included in the article/supplementary material, further inquiries can be directed to the corresponding author.
